# Air Embolism: Diagnosis, Clinical Management and Outcomes

**DOI:** 10.3390/diagnostics7010005

**Published:** 2017-01-17

**Authors:** Colin J. McCarthy, Sasan Behravesh, Sailendra G. Naidu, Rahmi Oklu

**Affiliations:** 1 Massachusetts General Hospital, Harvard Medical School, Division of Interventional Radiology, 55 Fruit Street, GRB-290A, Boston, MA 02114, USA; colin.mccarthy@mgh.harvard.edu; 2 Mayo Clinic, Division of Vascular and Interventional Radiology, 13400 E Shea Blvd, Scottsdale, AZ 85259, USA; sasan.behravesh@gmail.com (S.B.); naidu.sailen@mayo.edu (S.G.N.)

**Keywords:** air embolus, angiography, embolization

## Abstract

Air embolism is a rare but potentially fatal complication of surgical procedures. Rapid recognition and intervention is critical for reducing morbidity and mortality. We retrospectively characterized our experience with air embolism during medical procedures at a tertiary medical center. Electronic medical records were searched for all cases of air embolism over a 25-year period; relevant medical and imaging records were reviewed. Sixty-seven air embolism cases were identified; the mean age was 59 years (range, 3–89 years). Ninety-four percent occurred in-hospital, of which 77.8% were during an operation/invasive procedure. Vascular access-related procedures (33%) were the most commonly associated with air embolism. Clinical signs and symptoms were related to the location the air embolus; 36 cases to the right heart/pulmonary artery, 21 to the cerebrum, and 10 were attributed to patent foramen ovale (PFO). Twenty-one percent of patients underwent hyperbaric oxygen therapy (HBOT), 7.5% aspiration of the air, and 63% had no sequelae. Mortality rate was 21%; 69% died within 48 hours. Thirteen patients had immediate cardiac arrest where mortality rate was 53.8%, compared to 13.5% (*p* = 0.0035) in those without. Air emboli were mainly iatrogenic, primarily associated with endovascular procedures. High clinical suspicion and early treatment are critical for survival.

## 1. Introduction

Iatrogenic procedures are the main cause of vascular air embolism (VAE). This rare complication can arise in a wide range of clinical scenarios involving line placement, trauma, barotrauma, and several types of surgical procedures including cardiac, vascular, and neurosurgery. Traditionally, surgery and trauma were the most significant causes of systemic and cerebral air embolism; however, endoscopy, angiography, tissue biopsy, thoracocentesis, hemodialysis, and central/peripheral venous access now comprise a greater proportion [1,2,3]. The insertion and maintenance of advanced vascular access devices are increasingly being performed within multiple clinical specialties. Moreover, the bulk of interventional radiology (IR) procedures commence with the placement of an intravascular sheath, which is a major risk factor for air embolism throughout the duration of the procedure. Endovascular procedures complicated by an intravascular air embolism result in significant morbidity and mortality. VAE is a potentially preventable condition, which arises as a result of a pressure gradient that allows air to enter the blood stream, which can subsequently cause blockages in blood flow. VAE has an estimated incidence of 1 in 772 according to one series, while another study found that the incidence of iatrogenic gas embolism complicates 2.65 per 100,000 hospitalizations; however, these figures are considered lower than the true incidence due to many unreported instances and undiagnosed asymptomatic patients [4,5,6]. In this article, we set out to review all cases of air embolism in a tertiary medical center, including analysis of cause, clinical signs and symptoms, treatment, and prognosis.

## 2. Materials and Methods

Institutional Review Board approval was obtained for this retrospective single-center study. Electronic medical records were searched for all cases of air embolism over a 25-year period using the ICD-9-CM code for air embolism; 958.0. Furthermore, a separate database of radiology reports was searched for the terms “air embolism”, “air embolus”, and other related terms over the same time period. The registry data was retrieved, combined, and subsequently, the relevant medical and imaging records were reviewed. Patients with imaging evidence or high clinical suspicion of air embolism were identified. Inclusion criteria included the presence of sudden desaturation, reduced end-tidal CO_2_, acute cardiopulmonary compromise as defined by the need for cardiopulmonary resuscitation or vasopressors, acute neurological change including motor or sensory changes, or the presence of seizures. Additionally, all patients needed to have a clearly defined antecedent event, including open, laparoscopic, or endovascular procedures, central or peripheral line placement or removal, or an environmental cause such as SCUBA (self-contained underwater breathing apparatus) diving. Variables including location of the event, immediate signs/symptoms, outcome at discharge, and treatments utilized were recorded and analyzed. Statistical analysis to assess for differences between patient groups was performed using Fisher’s exact test.

## 3. Results

Sixty-seven patients were identified to have had an air embolism over the study period: 34 male and 33 female patients, with a mean age of 59 years (range 3–89 years). The majority of cases (94%, *n* = 63) occurred within the hospital, with four cases occurring in the community (Table 1). Of those cases occurring within the hospital, the most common setting was during a surgical procedure (77.8%, *n* = 49), including those occurring in interventional radiology (IR). Of these 49 cases, 33 occurred in the operating room, nine occurred in the IR suite, four in the cardiac catheterization laboratory, two cases in the endoscopy room, and one in the perioperative area. Of those four cases occurring in the community, one was related to SCUBA and near-drowning, and one other was related to snorkeling.

The intraoperative cases included a wide variety of procedures, ranging from vascular access to open neurosurgical procedures (Table 2). The most common type of procedures associated with air embolism were central vascular access (*n* = 9) and open neurosurgical procedures (*n* = 11). Of the vascular access cases, four occurred at the time of dialysis line placement, two during central line placement, one during a Hickman line placement, and two during the placement of a chest wall port. Of the open neurosurgical cases, six occurred during emergency procedures following trauma and/or acute intracranial hemorrhage, and five occurred during elective procedures. Thirty-four percent (*n* = 23) of patients had premortem evidence of air embolism on imaging studies.

**Figure 1:**
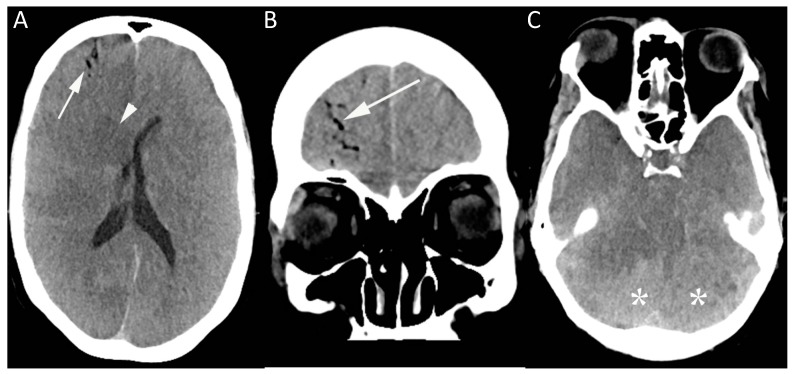
This image shows a computed tomography (CT) brain of a 50-year-old female in the medical intensive care unit who became unresponsive shortly after removal of an internal jugular line. CT brain (**A**–**C**) was performed showing gas within the right frontal lobe in distribution, suggesting an intravascular location (arrow, **A** and **B**), together with diffuse loss of gray-white matter differentiation, most prominently in the central deep nuclei (arrowhead). There was evidence of mass effect, with effacement of the basal cisterns (**C**), and the dense cerebellum sign (*) related to relative sparing of the cerebellar hemispheres.

When all locations were included, vascular access-related procedures were most commonly associated with air embolism. In addition to the nine cases related to line placement described above, 13 cases were noted to have occurred during the use or removal of vascular access (Figure 1; Table 3), including two cases of inadvertent air bolus administered via intravenous lines.

There were 36 cases of an air embolism to the right heart or pulmonary artery, and 21 cases of cerebral embolism, with the remainder of cases involving the extremities or coronary arteries (Table 4). There were 14 cases of suspected paradoxical air embolism, when air from the venous system reached the arterial system through a right to left shunt, most commonly a patent foramen ovale (PFO). Thirteen cases were associated with paradoxical embolus to the cerebral circulation, and one case involved paradoxical embolus to a coronary artery across a PFO at the time of central line removal. Echocardiography was performed in all but one of these patients, and a PFO was identified in 77% of cases (*n* = 10).

Immediate clinical signs and symptoms were related to the location to which the air embolus had traveled; for example, cerebral air embolism was associated with neurological signs including weakness and seizures (Table 5). Immediate cardiac arrest occurred in 13 patients. In a minority (15%, *n* = 10) of cases, the patients were found to be entirely asymptomatic, despite clear antecedent event and imaging or clinical evidence of intravascular air.

Treatment of air embolism included hyperbaric oxygen therapy (HBOT) in 14 cases and extracorporeal membrane oxygenation (ECMO) in two cases. The majority of patients (54%, *n* = 46) were treated in an intensive care unit (ICU), although in many cases, this was not necessarily related to the air embolism alone, but concomitant medical conditions. Aspiration of the air embolism was attempted in five cases, with four of these patients being discharged with no sequelae and one patient dying.

**Figure 2:**
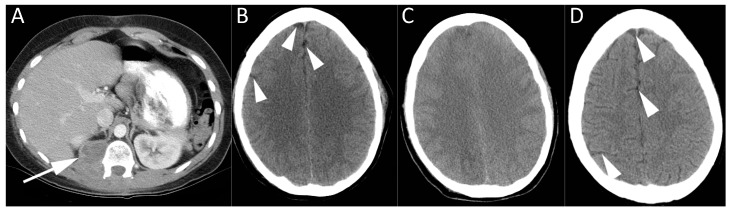
CT imaging of a 20-year-old female with paraspinal tumor seen on axial contrast enhanced CT (**A**). She became profoundly hypotensive during spinal surgery for resection of this malignant nerve sheath tumor. Air was noted in an infusion bag. CT brain at baseline (**B**) was normal, revealing normal volume of cerebrospinal fluid (CSF) around the cerebral hemispheres (arrowheads). Serial CT studies of the brain performed over the coming days revealed progressively worsening cerebral edema. CT brain at three days (**C**) shows effacement of CSF and diffuse cerebral swelling. She was managed with hyperbaric oxygen treatment and an intracranial pressure monitor was placed. CT brain performed 19 months later showed essentially normal appearances of the brain (**D**), with return of normal CSF spaces (arrowheads). The patient recovered from the air embolism, but died 25 months later from metastatic disease.

Forty-two patients (63%) were discharged from the hospital with no sequelae. Of the 23 patients who suffered an acute neurological change (Table 5), four died, ten were discharged with residual deficits, and nine were discharged with no residual deficit (Figure 2). Fourteen deaths occurred, with the majority of patients (69%, *n* = 11) dying within 48 h (Table 6). In 9 out of the 14 deaths, contributory factors were present, including severe head trauma (*n* = 5), severe coronary artery disease (*n* = 1), recent acute myocardial infarct requiring coronary artery bypass grafting (CABG) (*n* = 1), respiratory failure from lung abscess/pneumonia (*n* = 1), and severe anoxic brain injury related to near-drowning (*n* = 1).

The overall mortality rate was 21%. The mortality rate in those with cardiac arrest as an initial clinical presentation was 53.8%, compared to those who did not suffer cardiac arrest, where the mortality rate was 13.5%. This difference was found to be statistically significant (*p* = 0.0035).

## 4. Discussion

Air embolism may cause end-organ ischemia or infarction if there is insufficient collateral supply. Air emboli to the coronary or cerebral circulation can have major adverse consequences, even when the volume of air is small. A venous gas embolism (VGE) occurs when air enters the venous system and eventually causes an obstruction in the pulmonary circulation. This can arise as a result of a trauma or from a multitude of iatrogenic procedures. An anatomic cardiac defect—or in certain conditions, oxygen toxicity and excessive volumes of gas—can lead to the passage of air bubbles through the pulmonary vasculature. The gradient between external atmospheric pressure and the intravascular central venous pressure (CVP) is especially increased by hypovolemia or during inspiration by creating a negative intrathoracic pressure, which can enhance the possibility of air entry. As CVP may be sub-atmospheric at baseline in up to 40% of patients [7], those patients in an upright position or those undergoing IR procedures such as hemodialysis catheter placements are particularly susceptible.

In contrast, arterial gas embolism (AGE) forms when air or gas enters the arterial circulation. This type of air embolism can form upon the direct instillation of air into the arterial tree (i.e., angiography) or paradoxically, through a septal defect or patent foramen ovale (PFO). In our series, a PFO was detected in 77% of cases where an echocardiogram was performed for suspected paradoxical air embolism, compared to an estimated incidence of about 9% in the general population [8].

The clinical signs and symptoms recorded related to the mechanism of air entrapment and the location of the air embolism. Neurological symptoms may occur in air embolism, both due to direct passage of air to the cerebral circulation and related to reduced cardiac output secondary to circulatory collapse. AGE often becomes apparent with a range of symptoms akin to that of a stroke, including focal neurological deficits. Seizures, loss of consciousness, confusion, altered mental status, and paralysis has also been observed. In general, symptoms and signs associated with serious air embolism are predominantly non-specific. Diagnosis of air embolism can often be missed when dyspnea, continuous coughing, chest pain, and a sense of “impending doom” make up the chief clinical symptoms. Corresponding clinical signs include cyanosis, hypoxia, hypercapnia, hypotension, tachypnea, wheezing, bronchospasm, tachycardia, or bradycardia [9]. Pulmonary edema and an AGE due to a transpulmonary passage or right to left shunt through a PFO can be caused by a VGE. Small amounts of VGE are often tolerated and asymptomatic in patients, as they are absorbed due to filtration by pulmonary capillary beds.

Arterial obstruction or endothelial damage and secondary vasospasm and capillary leak can be caused by intravascular gas. Furthermore, according to a case report, air embolism has been noted as the cause of endothelium-derived cytokine release, leading to initiation of the systemic inflammatory response [10].

The degree of morbidity and mortality in venous air emboli are associated with the volume of gas, the rate of accumulation, and the patient’s position at the time of the event. The estimated adult lethal dose of air has been estimated at between 200 and 300 mL (3–5 mL/kg) [1], an amount which can be introduced in just 2–3 s with a 14-gauge needle and a pressure gradient of 5 cm H_2_O [11]. Essentially, the closer the air entry is to the right heart, the less volume of air is required to have fatal consequences.

As a result, it follows that many open and percutaneous procedures involving the vascular system are associated with potential risk of air embolism. For example, as mentioned previously, vascular sheaths are often used in IR to facilitate placement of wires and catheters, offering a potential route for the introduction of intravascular air. The risk is also present in open surgical procedures; for example, in neurosurgery, where patients may be operated on in an upright position, resulting in low pressure in the dural venous sinuses, with estimated rates of air embolism ranging from 10% to 80% [12,13]. Arterial air embolism may be due to direct instillation of air into the arterial tree (for example, during angiography), or be paradoxical, related to a venous air embolus that has crossed to the left heart through a PFO.

To our knowledge, this is the largest case series looking at all causes of air embolism. These 67 patients represent a diverse group with a variety of underlying medical conditions. However, many of the patients in our series had undergone invasive medical procedures known to be associated with a risk of air embolism, including central vascular line placement [14] and removal [15], bronchoscopic Nd:YAG laser procedures [16], together with neurosurgery [1,17] and cardiac surgery [18].

**Figure 3:**
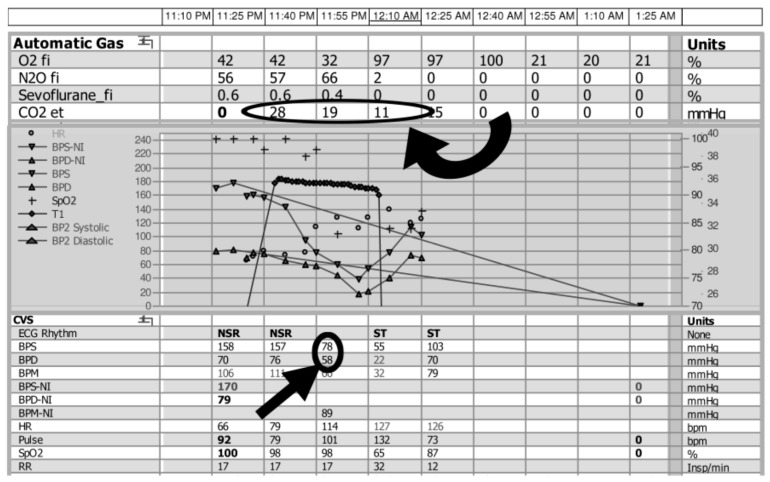
Changes in end-tidal CO_2_ monitoring during intraoperative air embolism from the anesthesia record of a 76-year woman undergoing hemicraniectomy for traumatic intracranial hemorrhage. Approximately 5 min after the cranium was opened, a sudden decrease in end-tidal CO_2_ was noted (circled, curved arrow) followed by a rapid decrease in both the systolic (BPS) and diastolic (BPD) blood pressure (circled, straight arrow). Immediate measures were taken, including lowering the head and flooding the field with water. However, 3 min after the change in CO_2_ was detected, the patient suffered a pulseless electrical activity (PEA) cardiac arrest, and expired 1 h later despite resuscitative measures.

When air reached the right ventricle or pulmonary artery, there was evidence of desaturation with varying levels of cardiopulmonary compromise, ranging from hypotension to cardiovascular collapse. In many patients who were under anesthesia, the first clinical change noted was a sudden decrease in end-tidal CO_2_ (Figure 3). Although pulse oximetry provides details on the level of blood oxygenation, end-tidal CO_2_ monitoring allows for evaluation of the effectiveness of ventilation, by either graphically (capnography) or numerically (capnometry) recording the CO_2_ being eliminated from the respiratory system. In general, as with carbon dioxide measurement, it is a less sensitive measurement than many alternative options. In this case series, a decrease in end-tidal CO_2_ was reported as an initial sign in 12 patients. Although such monitoring is performed routinely in patients under general anesthesia, it has recently been recommended for use during cases utilizing moderate and deep sedation, of particular relevance to the interventional radiology community [19,20,21].

In addition to supportive treatment including the use of 100% oxygen, immediate treatment options when air embolus is suspected include closing off any conduit between the atmosphere and the vascular system. Aspiration of the air should be attempted, for example, if there is a pre-existing indwelling catheter. Although de novo placement of a vascular catheter purely for the purpose of attempting to aspirate air is somewhat controversial [1], there are certainly reports of success using this technique [22]. In our series, aspiration was attempted in at least five cases. In cases of venous air embolism, Durant’s maneuver may be performed [23]; by placing the patient in the left lateral decubitus and Trendelenberg position, this serves to encourage the air bubble to move out of the right ventricular outflow tract (RVOT) and into the right atrium, thereby relieving the “air-lock” effect responsible for potentially catastrophic cardiopulmonary collapse.

Hyperbaric oxygen therapy plays a key role in the treatment of air embolism. It has been shown that the size of the air bubble is inversely proportional to the atmospheric pressure, based on the relationship between pressure and volume in a gas. For example, at six atmospheres of pressure, the relative volume of a gas bubble is 17% that at atmospheric pressure [24]. In our cohort, 14 patients were treated with hyperbaric oxygen therapy (HBOT), with the percentage mortality in that group being 14.3%, compared to 22.6% of patients who died in the group that did not receive HBOT. However, this difference was not statistically significant (*p* = 0.72). Currently, HBOT is not administered routinely to all patients with air embolism, as it may not always be indicated and its appropriateness must be assessed, bearing in mind the potential detriments involved in administering it (transportation and the length of time in the HBOT chamber) [25,26,27,28]. Evidence suggests that when indicated, it must be stressed that HBOT ideally is started within the first four to six hours after onset of neurologic symptoms, and also at any manifestation of end-organ damage, cardiopulmonary, or hemodynamic compromise [29,30]. Once the need for definitive therapy which includes HBOT has been identified, institution of such therapy should be expedited. Some studies indicate that HBOT treatment can still have a beneficial role up to 30 h after the initial event [31,32], but some authors have raised the possibility that not all physicians are enthusiastic about hyperbaric treatment for air embolism, despite several series showing good outcomes if HBOT is commenced at an early stage [33].

Despite being rare, as a result of its high chance of mortality and morbidity, it is imperative that physicians are capable of recognizing and dealing with air embolism. Prior to the early 1970s, air embolism after trauma was essentially unrecognized; since then, numerous case reports and series have been published. However, the amount of available literature is still small; this can partly be attributed to under- and misdiagnosis, as a cerebral insult will often be linked to other causes [34,35,36]. Systematic planning, prompt recognition, and focused treatment with supplemental O_2_ administration and ideally hyperbaric oxygen therapy offer the best chance of survival when indicated. Even with appropriate treatment, recent figures suggest that the overall one-year mortality may be approximately 20%—a finding similar to our study, where the mortality rate in the entire cohort was 21% [5]. However, it should be noted that our study addressed those patients for whom air embolism was diagnosed clinically or on the basis of imaging, and this may preferentially identify more serious cases of air embolism. As noted above, there are likely many cases of air embolism that go undetected due to an absence of clinical signs and/or symptoms.

## 5. Conclusions

In conclusion, the majority of cases of air embolism were iatrogenic, most commonly related to invasive medical procedures, with environmental causes such as those related to trauma and SCUBA diving representing a small minority of cases. Cardiac arrest immediately following air embolism is associated with a higher risk of mortality. High clinical suspicion to allow for early recognition and treatment is critical. Advances in technology and updated guidelines suggest that consideration should be given to utilizing end-tidal CO_2_ monitoring, even in non-intubated patients undergoing procedures. Principles from fields outside of medicine may be advantageous to incorporate. Checklists in aviation are commonplace, but have been infrequent in the surgical suite until recently [37]. To our knowledge, checklists for air embolism prevention do not exist, though there could be evidence to support their use [37]. Air embolism is listed as a “never event” by The National Quality Forum, highlighting the importance of instituting effective preventative and management measures [38]. Ultimately, institutional risk-reduction policies should at a minimum be established, and the implementation of standardization, staff training, and targeted requisition of equipment with anti-embolism safety features is crucial [33,39].

## Figures and Tables

**Table 1:** Location of events resulting in air embolism.

Location of Event	Number of Patients
Community	4
Dialysis	2
Floor/ICU (intensive care unit)	11
Intraoperative (including interventional radiology)	48
Peri-operative	1
Radiology (excluding interventional radiology)	1

**Table 2:** Intraoperative/intra-procedural cases associated with air embolism.

Procedure	Number
Abdominal aortic aneurysm repair	1
Abdominoperineal resection	1
Atrial septal defect closure	2
Bronchoscopy	1
Coronary artery bypass grafting	2
Cardiac ablation	1
Cardiac catheterization	3
Inadvertent air bolus via IV line	1
Central vascular access	9
Endoscopic Retrograde Cholangio-Pancreatography (ERCP)	1
Hysteroscopy	1
Laparoscopic liver resection	2
Necrotic bowel resection	1
Endovascular neurointervention	2
Neurosurgery (open procedures)	11
Pacemaker placement	1
Spinal surgery	2
Transarterial chemoembolization (TACE)	1
Cardiac valve replacement	4
Varicose vein injection	2

**Table 3:** Cases of air embolism occurring related to the use, placement, or removal of peripheral or central venous access.

Central Vascular Access	Number	Total
Central line before hip surgery	1	9
Dialysis catheter placement	4	
Hickman placement	1	
Central line placement during management of polytrauma	1	
Port placement	2	
**Central line removal**		
Removal of internal jugular line	5	7
Removal of pulmonary arterial line	1	
Removal of subclavian line	1	
**Other line-related**		
Patient absconded from hospital and removed central line at home	1	6
Inadvertent bolus of air via venous access device	2	
Patient removed hubs from peripherally inserted central catheter (PICC) at home	1	
Peripheral IV line placement	2	

**Table 4:** Anatomic location of air embolism.

Anatomical Location	Number
Coronary artery	8
Lower extremity	1
Pulmonary artery/right heart	36
Cerebral	21
Pulmonary artery/right heart and cerebral arteries	1

**Table 5:** Immediate clinical signs and symptoms associated with air embolism.

Immediate Clinical Signs/Symptoms	Number
Acute myocardial infarct	1
Bradycardia, hypotension, unresponsive	1
Cardiac arrest	13
Desaturation	5
Desaturation and hypotension	3
Desaturation and neurological signs/symptoms	3
Desaturation, reduced end tidal carbon dioxide (ETCO_2_)	6
Desaturation, reduced ETCO_2_, and hypotension	6
Neurological signs/symptoms, without seizure	16
None observed clinically or seen on imaging *	10
Seizure	3

* Imaging includes fluoroscopy, computed tomography, or echocardiography.

**Table 6:** Patient deaths related to air embolism.

Time to Death	Number
<24 h	5
24–48 h	6
3–7 days	2
>7 days *	1

* Patient expired 59 days following central line removal at outside hospital with resultant catastrophic cerebrovascular accident.
